# Associations between maternal overweight/obesity during pregnancy and body composition in young adult offspring

**DOI:** 10.3389/fpubh.2024.1346900

**Published:** 2024-03-13

**Authors:** Amaraporn Rerkasem, Jaz Lyons-Reid, Sirianong Namwongprom, Suthathip Wongsrithep, Ampica Mangklabruks, Kochaphan Phirom, Kittipan Rerkasem, José G. B. Derraik

**Affiliations:** ^1^Environmental-Occupational Health Sciences and Non-Communicable Diseases Research Group, Research Institute for Health Sciences, Chiang Mai University, Chiang Mai, Thailand; ^2^Liggins Institute, University of Auckland, Auckland, New Zealand; ^3^Department of Radiology, Faculty of Medicine, Chiang Mai University, Chiang Mai, Thailand; ^4^Department of Internal Medicine, Faculty of Medicine, Chiang Mai University, Chiang Mai, Thailand; ^5^Department of Surgery, Faculty of Medicine, Clinical Surgical Research Center, Chiang Mai University, Chiang Mai, Thailand; ^6^Department of Women’s and Children’s Health, Uppsala University, Uppsala, Sweden; ^7^Department of Paediatrics: Child and Youth Health, Faculty of Medical and Health Sciences, University of Auckland, Auckland, New Zealand

**Keywords:** adiposity, birth cohort, body fat, dual-energy X-ray absorptiometry, DXA, follow-up

## Abstract

**Background:**

Maternal obesity is associated with an increased risk of large-for-gestational-age births and childhood obesity. However, evidence on its potential associations with long-term offspring body composition remains limited. This prospective cohort study examined associations between maternal body mass index (BMI) during pregnancy and body composition in the young adult offspring.

**Methods:**

Participants were the offspring from a birth cohort in Chiang Mai (Thailand). Maternal BMI was assessed at the first antenatal clinic visit (≤24 weeks of gestation) in 1989–1990. In 2010–2011, we followed up the offspring at approximately 20 years of age, assessing their body composition using whole-body dual-energy X-ray absorptiometry (DXA) scans. Associations between maternal BMI and offspring body composition were explored using unadjusted and adjusted analyses.

**Results:**

We assessed 391 young adults (55% were females). Higher maternal BMI was associated with increased offspring fat mass and lean mass. In adjusted analyses, offspring of mothers with overweight/obesity exhibited total body fat percentages 1.5 (95% CI 0.1, 2.9; *p* = 0.032) and 2.3 (95% CI 0.2, 4.5; *p* = 0.036) percentage points higher than offspring of normal-weight and underweight mothers, respectively. Fat mass index was similarly higher: 0.9 kg/m^2^ (95% CI 0.3, 1.5 kg/m^2^; *p* = 0.002) and 1.4 kg/m^2^ (95% CI 0.5, 2.3 kg/m^2^; *p* = 0.002), respectively. However, no differences in visceral adiposity were detected.

**Conclusion:**

Higher maternal BMI during pregnancy was associated with increased adiposity in young adult offspring. Our findings suggest that the cross-generational transmission of maternal obesity-related traits is associated with increased offspring adiposity in the long term.

## 1 Introduction

Higher maternal body mass index (BMI) is associated with increased risks of large-for-gestational-age births and childhood obesity (1–3), as well as increased adiposity in the offspring (4). Although associations between maternal obesity and offspring health outcomes are well-documented, evidence regarding increased obesity risk in adult offspring primarily derives from conventional anthropometric measurements, such as waist circumference, weight, height, waist-to-hip and waist-to-height ratios, and BMI (3, 5). Thus, the potential impact on body composition in adult offspring is not well-defined.

Dual-energy X-ray absorptiometry (DXA) is a reliable method for assessing body composition, which can estimate whole-body and regional fat mass (FM), lean mass (LM), and bone mineral density (BMD) (6, 7). Some DXA scanners can also estimate visceral adipose tissue (VAT), which is known to be associated with metabolic risk (6). Therefore, DXA enables the measurement of whole-body FM and FM distribution, both important predictors of cardiometabolic outcomes in adulthood (4). We have previously reported an association between increased body fat (estimated with DXA) and metabolic syndrome in young Thai adults (8). However, the evidence linking maternal obesity to obesity risk and cardiometabolic health in adult offspring is predominantly from developed countries (9). Such evidence may not be directly extrapolated to Thailand due to distinct genetic, cultural, lifestyle, and environmental factors that can influence obesity risk differently compared to Western populations (10). However, in recent decades, Thailand has experienced a shift from an agricultural to a more industrialized economy and from rural to urban living, leading to marked changes in dietary habits and physical activity levels (11, 12). This shift has escalated overweight and obesity prevalence in the country (13).

In 1989–1990, the rates of overweight and obesity among more than 2,000 pregnant women in Northern Thailand were 13.9 and 10.4%, respectively (14), based on the Asia-Pacific BMI cut-offs of ≥23 to <25 kg/m^2^ and ≥25 kg/m^2^, respectively (10). A later study in 2006–2007 employing the same Asia-Pacific BMI criteria reported higher rates, with 22.4% of pregnant women having overweight and 10.1% obesity (15).

According to the most recent data from the World Obesity Federation (16) based on the World Health Organization (WHO) BMI classification (17)—which defines overweight as BMI ≥25 to <30 kg/m^2^, and obesity as a BMI ≥30 kg/m^2^—the combined prevalence of overweight and obesity among Thai adults has been increasing at a rate of 5.3% per annum, with higher rates in women compared to men (16). Using the same WHO criteria, Thailand has one of the highest obesity rates for women of reproductive age among the 10 member countries of the Association of Southeast Asian Nations (ASEAN), second only to Malaysia (16, 18).

Obesity in pregnancy is associated with an increased risk of adverse pregnancy and birth outcomes, and is a strong predictor of obesity in the offspring (19, 20). Therefore, the increasing rates of obesity among Thai adults are concerning, particularly among women of reproductive age. However, in Thailand, there remains a lack of data on the impact of maternal obesity during pregnancy on long-term offspring health. Addressing this data gap is important for developing targeted interventions aimed at reducing the long-term adverse health consequences of obesity in the Thai population. Thus, we assessed the associations between maternal BMI and body composition in their young adult offspring.

## 2 Methods

### 2.1 Study population

In this study, we analyzed data from a longitudinal, long-term birth cohort in Chiang Mai, Northern Thailand, which recruited 2,184 women carrying singletons in 1989–1990 (14). The original study required participants to meet the following inclusion criteria:

1. Be registered for prenatal care at either Maharaj Nakorn Chiang Mai Hospital or Chiang Mai Medical Center Hospital; AND.
2. Be carrying a single fetus (singleton pregnancy); AND.
3. Be enrolled in the study at ≤24 weeks of gestation.

Participants were excluded if they experienced miscarriage, stillbirth, gave birth at a non-participating facility, or if essential birth information could not be acquired (14).

In 2010–2011, a follow-up study was conducted on the offspring (who were approximately 20 years old) from the original cohort (21). Many families were unreachable, while others declined to participate or failed to attend the clinical assessments; therefore, we studied 632 young adults (21) (Supplementary Figure S1). Previously, we reported associations between maternal overweight/obesity and greater BMI and increased obesity risk in the 632 offspring (21). In this study, we report body composition data on a subset of 391 young adults who also underwent a whole-body dual-energy X-ray absorptiometry (DXA) scan (Supplementary Figure S1).

### 2.2 Clinical assessments

In the original study, maternal weight and height were measured at the first antenatal clinic visit (14), occurring at a median of 14 weeks of gestation [Q1 = 11, Q3 = 18 weeks] (Supplementary Figure S2). Their early to mid-pregnancy BMI (hereafter referred to as pBMI for greater clarity) was subsequently calculated. Of note, pBMI and birth weight were similar among mothers assessed at ≤14 weeks of gestation compared to those assessed at >14 weeks (Supplementary Table S1).

Additional data of interest were extracted from study records. Demographic characteristics included maternal age (at the first antenatal visit), family income, and maternal and paternal highest levels of education. Clinical data included parity and smoking during pregnancy, and birth outcomes, namely delivery type (vaginal or cesarean section), offspring sex, birth weight, and gestational age at birth. The offspring underwent anthropometric assessments (i.e., waist circumference, height, and weight) at the 20-year follow-up at the Maharaj Nakorn Chiang Mai Hospital, with participants barefoot and in light clothing. During face-to-face interviews, we collected information on the participant’s medical history, including medication use.

Maternal and offspring BMI were calculated and categorized according to the Asia-Pacific cut-offs as follows: underweight (<18.5 kg/m^2^), normal weight (≥18.5 to <23 kg/m^2^), overweight (≥23 to <25 kg/m^2^), and obesity (≥25 kg/m^2^) (10). This classification differs from the more widely used WHO criteria (17) but was chosen to acknowledge the distinct health risk profiles among Asian populations. There is evidence that at a given BMI, Asians experience higher risks of metabolic syndrome, cardiovascular disease, and other obesity-related non-communicable diseases in comparison to White Caucasians (10, 22, 23).

The offspring’s body composition was assessed using a Discovery-A DXA scanner (Hologic Inc., Bedford, MA, United States), and analyzed with software version 12.3 (8). The DXA scanner was calibrated daily according to the manufacturer’s instructions using a standard calibration phantom (8). The total and regional estimates of fat mass (FM) and lean mass (LM), were obtained. Regional adiposity measures included visceral adipose tissue (VAT) mass, android fat percentage, gynoid fat percentage, and the android-to-gynoid-fat ratio (A/G ratio, a marker of central adiposity) (24). The total body fat percentage (%BF), fat mass index (FMI), lean mass index (LMI), and visceral adipose tissue index (VATI) were calculated (24, 25).

### 2.3 Statistical analyses

Demographic and birth characteristics were compared using general linear models or non-parametric Kruskal-Wallis tests for continuous parameters, and Fisher’s exact tests for categorical parameters, as appropriate. General linear regression models were used to examine continuous associations between maternal pBMI and offspring body composition. Additional analyses were conducted stratified by pBMI status.

Multivariable models were also run, adjusting for confounders, including gestational age at birth (27), parity (28), maternal age (29), and offspring sex (30). Potential interactions between these confounders and maternal pBMI were explored. However, none were statistically significant, so no interactions were included in the final models.

Analyses were also carried out with the population stratified using the WHO BMI classification (17): underweight, BMI <18.5 kg/m^2^; normal weight, BMI ≥18.5 to <25 kg/m^2^; and overweight/obesity BMI ≥25 kg/m^2^.

Statistical analyses were performed using Stata version 16.1 (StataCorp, Texas, United States) and SPSS v28 (IBM Corp, Armonk, NY, United States). All tests were two-tailed, with a significance level set at *p* < 0.05. We did not adjust for multiple comparisons, reflecting the exploratory nature of this study (31). Data on study outcomes are reported as unadjusted (crude) and adjusted β-coefficients, means, and mean differences, and their respective 95% confidence intervals (CI).

## 3 Results

This study assessed 391 participants at approximately 20 years of age (range 18.6–21.8 years), of whom 55% were female (*n* = 215) and the majority (84%) had never smoked (Supplementary Table S2). Clinical and demographic characteristics of included and excluded offspring were largely similar, except that participants with DXA scans were approximately 2.3 kg lighter (*p* = 0.048) and had BMI 0.8 kg/m^2^ lower (*p* = 0.018) (Supplementary Table S2).

Mothers with overweight/obesity were 2.0 and 3.8 years older than those with normal weight or underweight, respectively (*p* < 0.001), and had lower rates of nulliparity (33, 61, and 85%, respectively, *p* < 0.001) (Table 1). Offspring birth weight progressively increased with greater maternal pBMI (Table 1). Participants born to mothers with overweight/obesity were 175 g (95% CI 74, 276 g; *p* = 0.001) and 330 g (95% CI 174, 484 g; *p* < 0.001) heavier than the offspring of normal weight or underweight mothers, respectively (Table 1). Other demographic, lifestyle, and birth characteristics were similar across the pBMI categories (Table 1).

**Table 1:** Demographic and clinical characteristics of study participants (offspring) and their parents.

			Maternal pBMI status^a^			
	Characteristics	Levels	Underweight	Normal weight	Overweight/obesity	
*n*			39 (10.0%)	270 (69.0%)	82 (21.0%)	–
Participants	Age (years)		20.6 ± 0.4	20.5 ± 0.4	20.5 ± 0.5	ns
	Sex	Male	18 (46.2%)	114 (42.2%)	44 (53.7%)	ns
		Female	21 (53.9%)	156 (57.8%)	38 (46.3%)	
	Birth weight (kg)		2,787 ± 490	2,942 ± 399	3,117 ± 390	**
	Birth weight *z*-score		−0.42 ± 1.18	−0.05 ± 0.96	0.37 ± 0.94	***
	Birth weight status	SGA	18 (46.2%)	64 (23.7%)	12 (14.6%)	**
		AGA	21 (53.8%)	199 (73.7%)	67 (81.7%)	
		LGA	nil	7 (2.6%)	3 (3.7%)	
	Gestational age (weeks)		39.0 ± 1.6	39.2 ± 1.6	39.3 ± 1.5	ns
	Smoking	Never smoked	35 (89.7%)	230 (85.2%)	64 (78.0%)	ns
		Former smoker	nil	15 (5.6%)	5 (6.1%)	
		Current smoker	4 (10.3%)	25 (9.3%)	13 (15.8%)	
	Birth type	Vaginal	3 (4.7%)	23 (8.5%)	10 (12.2%)	ns
		Cesarean section	36 (92.3%)	247 (91.5%)	72 (87.8%)	
Mother	pBMI (kg/m^2^)		17.6 [17.0, 18.0]	20.6 [19.6, 21.6]	24.4 [23.6, 25.8]	–
	Age at ANC (years)		24.3 ± 4.2	26.1 ± 4.3	28.1 ± 4.3	***
	Smoked during pregnancy		nil	2 (0.7%)	1 (1.2%)	ns
	Parity	Nulliparous	33 (84.6%)	165 (61.1%)	27 (32.9%)	***
		Parous	6 (15.4%)	105 (38.9%)	55 (67.1%)	
	Education^b^	Less than high school	20 (66.7%)	189 (80.4%)	63 (86.3%)	ns
		High school or greater	10 (33.3%)	46 (19.6%)	10 (13.7%)	
Father	Education^b^	Less than high school	18 (60.0%)	172 (73.2%)	52 (71.2%)	ns
		High school or greater	12 (40.0%)	63 (26.8%)	21 (28.8%)	
Family	Income (baht per month)^c^		3,200 [2,000, 4,500]	2,100 [1,500, 3,400]	3,220 [2,200, 5,000]	*

Data are the median [quartile 1, quartile 3], the; mean ± standard deviation, or *n* (%), as appropriate. AGA, appropriate-for-gestational-age (birth weight ≥ 10th to ≤ 90th percentile); ANC, first antenatal clinic visit; LGA, large-for-gestational-age (birth weight > 90th percentile); ns, not statistically significant; pBMI, pregnancy body mass index; SGA, small-for-gestational-age (birth weight < 10th percentile).

a BMI was recorded at the first ANC (median of 14 weeks of gestation) in the original study in 1989–1990; BMI status was defined according to the Asia-Pacific criteria (10): underweight, BMI < 18.5 kg/m^2^; normal weight, BMI 18.5 to <23.0 kg/m^2^; and overweight/obesity, BMI ≥ 23 kg/m^2^.

b There were missing data on the highest levels of maternal and paternal education and the available sample sizes for the three groups (i.e., Underweight, Normal weight, and Overweight/Obesity) were 30 (76.9%), 235 (87.0%), and 73 (89.0%), respectively.

c Income recorded at the time of maternal recruitment to the original study (1989–1990) and not adjusted for inflation.

*p < 0.05, **p < 0.01, and ***p < 0.001 for overall differences between the Maternal pBMI groups; *p*-values were derived from Fisher’s exact tests for categorical parameters, a non-parametric Kruskal-Wallis test for family income, and general linear models for other continuous variables.

As previously reported among the 632 follow-up participants (21), increasing maternal pBMI was associated with greater weight, waist circumference, and BMI in this offspring subgroup (Table 2). In both unadjusted and adjusted analyses, increasing maternal pBMI was associated with increased offspring FM, FMI, LM, and LMI (Table 2). Each 1 kg/m^2^ increase in pBMI was associated with an additional 390 g of FM (95% CI 140, 650 g; *p* = 0.003) in unadjusted and 460 g (95% CI 200, 720 g; *p* = 0.001) in adjusted analyses (Supplementary Table S3). After adjustment for confounders, greater maternal pBMI was associated with a %BF increase of 1.5 percentage points for every 5 kg/m^2^ rise in pBMI (95% CI 0.35, 2.60%; *p* = 0.009) (Supplementary Table S3). However, there were no observed linear associations with the A/G ratio (Supplementary Table S3).

**Table 2:** Anthropometry and body composition derived from DXA scans in the offspring (*n* = 391) from the Chiang Mai Low Birth Weight Study stratified by their mother’s BMI status in pregnancy using the Asia-Pacific BMI cut-offs.

	Unadjusted			Adjusted		
	Underweight	Normal weight	Overweight/obesity	Underweight	Normal weight	Overweight/obesity
*n*	39 (10.0%)	270 (69.0%)	82 (21.0%)	39 (10.0%)	270 (69.0%)	82 (21.0%)
Height (cm)	163.3 (160.8, 165.9)	163.4 (162.5, 164.4)	165.3 (163.5, 167.0)	163.3 (161.5, 165.2)	163.8 (163.1, 164.5)	164.2 (162.9, 165.5)
Weight (kg)	52.74 (48.81, 56.67)**	55.91 (54.42, 57.40)**	61.01 (58.30, 63.72)	52.39 (48.71, 56.07)**	56.14 (54.76, 57.51)**	60.43 (57.85, 63.00)
BMI (kg/m^2^)	19.6 (18.4, 20.8)***	20.8 (20.4, 21.3)**	22.3 (21.4, 23.1)	19.5 (18.3, 20.7)***†	20.8 (20.4, 21.3)**	22.3 (21.5, 23.2)
Waist circumference (cm)	77.1 (74.0, 80.1)**	78.9 (77.8, 80.1)**	82.6 (80.5, 84.7)	73.4 (70.4, 76.5)**	75.2 (74.0, 76.3)**	79.0 (76.9, 81.1)
Lean mass (kg)	38.84 (36.02, 41.67)**	40.32 (39.24, 41.32)**	43.92 (41.97, 45.86)	38.85 (36.91, 40.78)**	40.32 (39.58, 41.05)**	43.92 (42.58, 45.25)
Lean mass index (kg/m^2^)	14.54 (13.81, 15.27)***	15.10 (14.82, 15.37)***	16.17 (15.67, 16.67)	14.43 (13.85, 15.02)***	15.16 (14.94, 15.38)**	15.99 (15.58, 16.40)
Fat mass (kg)	14.23 (12.17, 16.29)*	15.68 (14.90, 16.46)*	17.48 (16.05, 18.90)	14.23 (12.26, 16.20)**	15.68 (14.93, 16.43)**	17.48 (16.12, 18.84)
Fat mass index (kg/m^2^)	5.41 (4.62, 6.20)*	5.97 (5.68, 6.27)	6.56 (6.02, 7.10)	5.37 (4.65, 6.09)**	5.90 (5.63, 6.17)**	6.81 (6.30, 7.31)
Total body fat (%)	26.5 (24.1, 28.9)	27.7 (26.8, 28.6)	27.9 (26. 3, 29.6)	26.6 (24.9, 28.3)*	27.4 (26.8, 28.1)*	28.9 (27.7, 30.1)
Android fat (%)	25.8 (23.0, 28.4)	27.4 (26.4, 28.3)	27.9 (26.2, 29.7)	25.8 (23.6, 28.0)*	27.1 (26.3, 27.9)	28.8 (27.2, 30.3)
Gynoid fat (%)	31.5 (28.8, 34.2)	32.3 (31.3, 33.4)	31.7 (29.8, 33.6)	31.7 (30.1, 33.3)	31.93 (31.4, 32.5)	32.9 (31.8, 34.0)
Android-to-gynoid-fat ratio	0.83 (0.78, 0.87)*	0.86 (0.84, 0.87)	0.88 (0.85, 0.91)	0.82 (0.78, 0.86)*	0.86 (0.84, 0.87)	0.88 (0.85, 0.91)
Visceral adipose tissue (g)	194 (163, 225)	197 (186, 209)	221 (199, 242)	190 (159, 221)	198 (187, 210)	220 (199, 242)
Visceral adipose tissue index (kg/m^2^)	0.073 (0.061, 0.084)	0.074 (0.070, 0.078)	0.082 (0.074, 0.089)	0.071 (0.060, 0.082)	0.074 (0.070, 0.078)	0.082 (0.074, 0.090)

BMI, body mass index; DXA, whole-body dual-energy absorptiometry.

Data are the means and respective 95% confidence intervals. Adjusted data were derived from general linear models adjusted for gestational age, sex, birth order, and maternal age.

BMI was recorded at the first antenatal visit (median of 14 weeks of gestation) in the original study in 1989–1990; BMI status was defined according to the Asia-Pacific criteria (10): underweight, BMI < 18.5 kg/m^2^; normal weight, BMI ≥ 18.5 to <23 kg/m^2^; and overweight/obesity, BMI ≥ 23 kg/m^2^.

*p < 0.05, **p < 0.01, and ***p < 0.001 for pairwise comparisons to the offspring of mothers with overweight/obesity.

^†^*p* < 0.05 for a pairwise comparison between the offspring of mothers with normal weight and underweight.

In stratified analyses by the Asia-Pacific BMI classification, after adjustment for confounders, offspring born to mothers with overweight/obesity had %BF that was greater by 1.5 and 2.3 percentage points than offspring of mothers with normal weight (95% CI 0.13, 2.91%; *p* = 0.032) or underweight (95% CI 0.15, 4.49%; *p* = 0.036), respectively (Table 2). Differences were also observed in FMI [+0.90 kg/m^2^ (95% CI 0.33, 1.48 kg/m^2^; *p* = 0.002) and + 1.43 kg/m^2^ (95% CI 0.54, 2.33 kg/m^2^; *p* = 0.002), respectively] (Table 2). Similar between-group differences were observed for lean mass parameters (Table 2). In addition, the offspring of mothers with overweight/obesity had android fat 3 percentage points greater than the offspring of underweight mothers (95% CI 0.25, 5.77%; *p* = 0.033), so that their A/G ratio was also 7.3% greater (*p* = 0.018) (Table 2). However, there were no observed differences in the parameters of visceral adiposity (Table 2).

The analyses using the WHO BMI criteria confirmed the observed between-group differences reported above under the Asia-Pacific classification (Table 3), underscoring the robustness of these findings across different BMI criteria. Further, there were differences in body composition (i.e., FM, FMI, LM, LMI, and %BF) between maternal pBMI groups in both unadjusted and adjusted models, as the magnitude and significance level of these differences were accentuated (Table 3). Moreover, using the WHO criteria, lower android fat % and gynoid fat % were detected in the young adult offspring of mothers with normal weight or underweight, irrespective of adjustment for confounders (Table 3).

**Table 3:** Anthropometry and body composition derived from DXA scans in the offspring (*n* = 391) from the Chiang Mai Low Birth Weight Study stratified by their mother’s BMI status during pregnancy based on the World Health Organization (WHO) criteria.

	Unadjusted			Adjusted		
	Underweight	Normal weight	Overweight/obesity	Underweight	Normal weight	Overweight/obesity
*n*	39 (10.0%)	320 (81.8%)	32 (8.2%)	39 (10.0%)	320 (81.8%)	32 (8.2%)
Height (cm)	163.3 (160.8, 165.9)	163.9 (163.0, 164.8)	163.7 (160.9, 166.5)	163.4 (161.5, 165.2)	163.9 (163.3, 164.5)	163.7 (161.6, 165.7)
Weight (kg)	52.74 (48.80, 56.68)***	56.49 (55.11,57.86)**	63.19 (58.84, 67.54)	52.50 (48.84, 56.17)***†	56.51 (55.25, 57.76)**	63.29 (59.28, 67.31)
BMI (kg/m^2^)	19.6 (18.4, 20.8)***†	20.9 (20.5, 21.3)***	23.4 (22.1, 24.7)	19.5 (18.3, 20.7)***†	20.9 (20.5, 21.3)***	23.4 (22.1, 24.7)
Waist circumference (cm)	77.1 (74.1, 80.1)**	79.3 (78.2, 80.3)**	85.1 (81.7, 88.4)	77.0 (73.9, 80.0)**	79.3 (78.2, 80.3)**	85.1 (81.7, 88.4)
Lean mass (kg)	38.85 (36.00, 41.70)*	40.88 (39.88, 41.87)	43.93 (40.79, 47.08)	38.85 (36.91, 40.78)***†	40.88 (40.20, 41.55)**	43.93 (41.80, 46.07)
Lean mass index (kg/m^2^)	14.54 (13.80, 15.27)**	15.23 (14.97, 15,49)**	16.47 (15.66, 17.28)	14.46 (13.87, 15.04)***†	15.23 (15.03, 15.44)***	16.53 (15.89, 17.17)
Fat mass (kg)	14.23 (12.18, 16.28)***	15.73 (15.02, 16.45)**	19.77 (17.51, 22.02)	14.23 (12.27, 16.20)***	15.73 (15.05, 16.42)**	19.77 (17.60, 21.94)
Fat mass index (kg/m^2^)	5.41 (4.63, 6.19)***	5.98 (5.70, 6.24)**	7.50 (6.64, 8.36)	5.40 (4.68, 6.11)***	5.98 (5.73, 6.22)***	7.48 (6.70, 8.27)
Total body fat (%)	26.5 (24.1, 28.9)*	27.5 (26.6, 28.3)*	30.6 (27.9, 33.3)	26.6 (24.9, 28.3)**	27.5 (26.9, 28.1)**	30.4 (28.5, 32.3)
Android fat (%)	25.8 (23.3, 28.3)*	27.2 (26.3, 28.1)*	30.5 (27.7, 33.2)	25.8 (23.6, 28.0)**	27.2 (26.5, 28.0)*	30.4 (28.0, 32.8)
Gynoid fat (%)	31.5 (28.8, 34.2)	32.0 (31.0, 32.9)	34.4 (31.4, 37.4)	31.7 (30.1, 33.3)*	32.0 (31.4, 32.5)*	34.1 (32.4, 35.8)
Android-to-gynoid-fat ratio	0.83 (0.78, 0.87)	0.86 (0.85, 0.88)	0.88 (0.83, 0.93)	0.82 (0.78, 0.86)*†	0.86 (0.85, 0.87)	0.89 (0.85, 0.93)
Visceral adipose tissue (g)	194 (163, 225)	200 (189, 211)	230 (196, 264)	190 (160, 221)	200 (190, 211)	232 (199, 266)
Visceral adipose tissue index (kg/m^2^)	0.073 (0.061, 0.084)	0.075 (0.071, 0.079)	0.087 (0.074, 0.099)	0.071 (0.060, 0.083)	0.075 (0.071, 0.079)	0.088 (0.075, 0.100)

BMI, body mass index; DXA, whole-body dual-energy absorptiometry.

Data are the means and respective 95% confidence intervals. Adjusted data were derived from general linear models adjusted for gestational age, sex, birth order, and maternal age.

BMI was recorded at the first antenatal visit (median of 14 weeks of gestation) in the original study in 1989–1990; BMI status was defined according to the WHO criteria (17): underweight, BMI < 18.5 kg/m^2^; normal weight, BMI ≥ 18.5 to < 25 kg/m^2^; and overweight/obesity BMI ≥ 25 kg/m^2^.

*p < 0.05, **p < 0.01, and ***p < 0.001 for pairwise comparisons to the offspring of mothers with overweight/obesity.

^†^*p* < 0.05 for pairwise comparisons between the offspring of mothers with normal weight and underweight.

As expected, maternal pBMI status was associated with an increased risk of overweight and obesity in the offspring (Supplementary Table S4). This association between maternal overweight/obesity and an increased risk of overweight and obesity in the young adult offspring reflected the associations reported for the follow-up cohort as a whole (21). Notably, this pattern was also observed in the offspring who did not undergo DXA scans (Supplementary Table S5).

## 4 Discussion

Our previous work showed that maternal overweight/obesity during pregnancy was associated with higher BMI and increased risk of overweight and obesity in young adult offspring in Thailand (21). This study contributes additional insights into the nature of these associations, in particular, higher maternal pBMI is associated with increased markers of adiposity in the offspring (e.g., FM, FMI, and %BF). Further, we showed using DXA scans that the young adult offspring born to mothers with overweight/obesity during pregnancy (using the Asia-Pacific BMI cut-offs) have increased FM, FMI, and %BF. Importantly, comparisons using the WHO BMI criteria (which singles out mothers at the upper end of the BMI spectrum) magnified those observations, showing differences in regional adiposity (android fat % and gynoid fat %). However, there were no detectable differences in visceral adiposity.

Our findings support existing evidence from indirect methods on the associations between maternal overweight/obesity and increased offspring FM (4), noting that none of the 20 studies included in Castillo-Laura’s systematic review and meta-analysis examined DXA-derived body fat in adult offspring. However, despite the increased waist circumference among the offspring of mothers with overweight/obesity, there were no observed differences in VAT or VATI. While DXA-derived VAT estimates are highly correlated with those derived from magnetic resonance imaging (MRI) (32) and computerized tomography (CT) (33), we acknowledge the inherent limitations of DXA compared to these imaging modalities. Considering these methodological constraints, the absence of observed differences in VAT and VATI warrants cautious interpretation. Nonetheless, our findings indicated greater abdominal adiposity (i.e., greater A/G ratio) in the offspring of mothers with overweight/obesity. Given that our participants were young adults, we speculate that differences in visceral adiposity, if present, may become more pronounced with age (34), highlighting the importance of longitudinal follow-up.

Our study had limitations, including the absence of information on dietary habits and physical activity levels, factors that can influence body composition. However, data on other potentially important confounders, such as family income and parental education, were available and did not account for the observed associations with pBMI. There were also no data on paternal BMI, but the evidence suggests maternal BMI is a stronger predictor of offspring obesity risk (35). Further, the rates of maternal overweight and obesity in our study cohort were relatively low, and it was necessary to collapse these two groups into one to ensure more robust analyses. Additionally, the timing of the mother’s first antenatal visit (when pBMI was derived) varied somewhat. However, there were no differences in pBMI or birth weight when participants with a visit at or before the median were compared to those above the median. This suggests the variability in first visit timing did not substantially influence the study findings. Another limitation was the lack of DXA data from 38% of eligible offspring. However, our sensitivity analyses showed the same associations between maternal pBMI and offspring BMI and obesity risk, yielding strong indication that our key findings on body composition were unaffected by their exclusion. Nonetheless, our reduced statistical power might have hindered our ability to detect statistically significant associations for parameters of visceral adiposity, or clearer findings on A/G ratio (a marker of central adiposity). Importantly, a key strength of this study is its novelty as, to our knowledge, this is the first report of long-term associations between maternal pBMI, overweight/obesity, and DXA-derived offspring body composition in a Thai adult population.

In conclusion, this study provides evidence that offspring born to mothers with overweight/obesity during pregnancy have increased adiposity in young adulthood. However, findings on measures of visceral/abdominal adiposity were inconclusive. Continued long-term follow-up of this cohort is essential to ascertain whether the observed associations between increased maternal pBMI and greater offspring adiposity persist into later life, potentially manifesting in overt metabolic conditions.

## Data availability statement

The study data cannot be made available in a public repository due to the conditions of the ethics approval, as consent was not obtained from study participants to make their confidential health data publicly available. However, the anonymised data upon which this manuscript was based could be made available to other investigators upon a bona fide request, provided all necessary approvals (including ethics approval) for the study protocol and statistical analysis plan are obtained. Any queries should be directed to KR (rerkase@gmail.com).

## Ethics statement

Ethics approval for the original Chiang Mai Low Birth Weight Study was granted by the Human Experimentation Committee at the Faculty of Medicine, Chiang Mai University. The 20-year follow-up study on the offspring received ethics approval from the Human Experimentation Committee at the Research Institute for Health Sciences (RIHES), Chiang Mai University (Project Number 17/52). This study was conducted in accordance with institutional guidelines, adhering to the Declaration of Helsinki (26) and complying with relevant national and international regulations for medical research. All study participants provided written informed consent, and their data were de-identified prior to analyses.

## Author contributions

AR: Conceptualization, Data curation, Methodology, Formal analysis, Supervision, Writing – original draft; JL-R: Methodology, Writing – original draft; SN: Conceptualization, Methodology, Formal analysis, Writing – review & editing; SW: Data curation, Formal analysis, Writing – review & editing; AM: Conceptualization, Methodology, Writing – review & editing; KP: Data curation, Formal analysis, Writing – review & editing; KR: Conceptualization, Methodology, Project administration, Supervision, Writing – review & editing; JGBD: Conceptualization, Methodology, Data Curation, Formal analysis, Supervision, Writing – original draft.
