# Supplementary material for: Associations between maternal overweight/obesity during pregnancy and body composition in young adult offspring
Source: Front Public Health. 2024 Mar 13;12:1346900. doi: 10.3389/fpubh.2024.1346900 (PMC10968890; doi:10.3389/fpubh.2024.1346900)
Supplement: Supplementary file 1 [file Data_Sheet_1.pdf]

Supplementary Material

Supplementary Figure 1. Diagram showing the flow of participants into the Chiang Mai Low Birth Weight Study (1989–1990) in Thailand, and the subsequent recruitment of offspring into the follow-up study on body composition using whole-body dual-energy X-ray absorptiometry (DXA) scans (2010–2011).

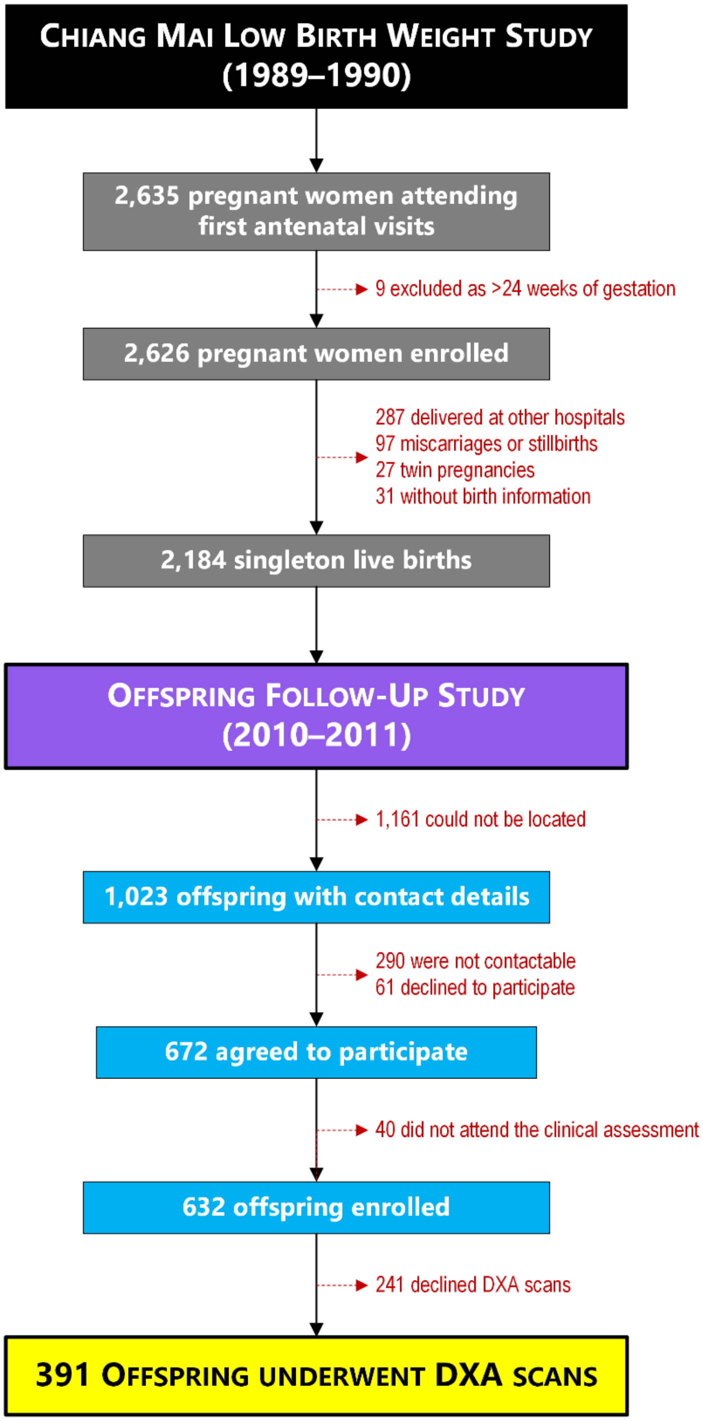

Rerkasem A, Lyons-Reid J, Namwongprom S, Wongsrithep S, Mangklabruks A, Phirom K, Rerkasem R, Derraik JGB. Associations between maternal overweight/obesity in early pregnancy and body composition in young adult offspring. *Frontiers in Public Health* 2024; 12: 1346900. doi: 10.3389/fpubh.2024.1346900

Supplementary Figure 2. Histogram showing the timing of the mothers' first antenatal care visit (ANC) in the Chiang Mai Low Birth Weight Study (1989–1990) in Northern Thailand, when anthropometric assessments were performed.

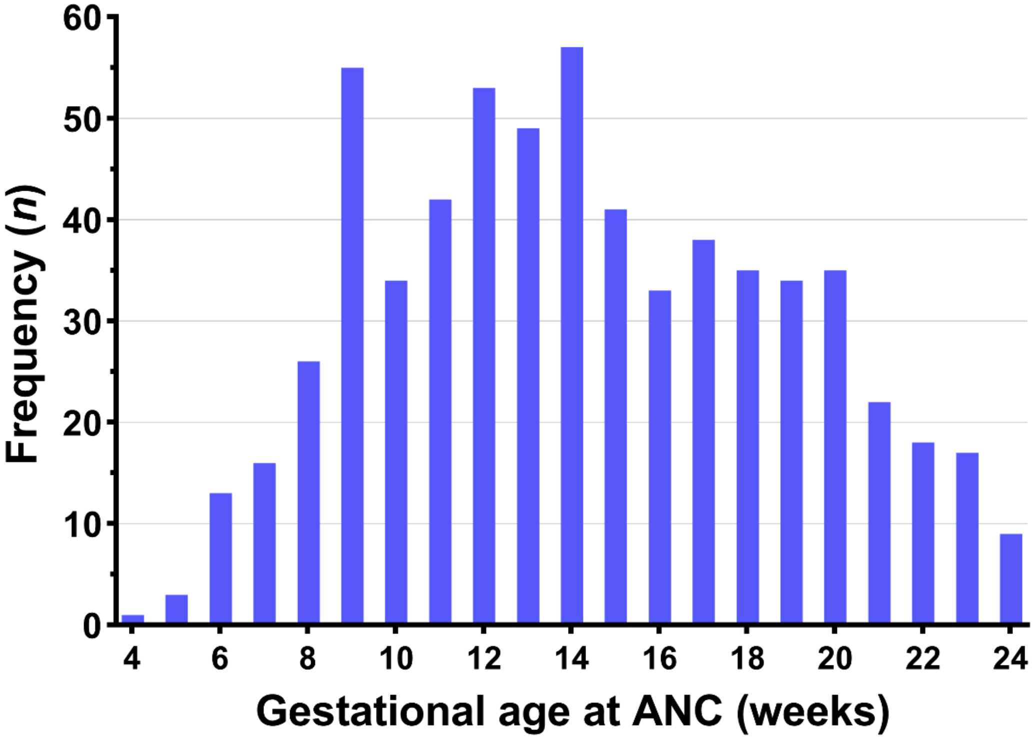

Supplementary Table 1. Maternal body mass index (BMI) at the first antenatal care visit (ANC) and offspring birth weight by ANC timing.

ANC Timing	≤14 weeks	>14 weeks	*p*-value
n	212	179	
**Maternal BMI**	21.1 ± 2.7	21.4 ± 2.3	0.34
**Birth weight (g)**	2972 ± 440	2953 ± 387	0.67
**Birth weight *z*-score**	0.02 ± 1.06	-0.02 ± 0.93	0.66

The two groups were stratified based on the median ANC timing (i.e., 14 weeks).
Data are the mean ± standard deviation.
p-values were derived from two-sample *t*-tests.

Supplementary Table 2. Maternal, birth, and adulthood characteristics of offspring from the Chiang Mai Low Birth Weight Cohort who did or did not undergo DXA scans.

Characteristic	Levels	With DXA (included)	Without DXA (excluded)
n		391	241
**Age (years)**		20.5 ± 0.5	20.7 ± 0.4 ***
**Sex**	**Females**	215 (55.0%)	126 (52.3%)
**Birth weight (kg)**		2963 ± 416	3004 ± 459
**Birth by caesarean section**		36 (9.2%)	21 (8.7%)
**Gestational age at birth (weeks)**		39.2 ± 1.6	39.2 ± 1.9
**Height (cm)**		163.8 ± 8.1	163.8 ± 8.6
**Weight (kg)**		56.7 ± 12.7	58.9 ± 15.8 *
**BMI (kg/m^2)**		21.0 ± 3.8	21.8 ± 4.9 **
**BMI status**	**Overweight/obesity**	93 (23.8%)	69 (29.0%)
**Smoking status**	**Never smoked**	329 (84.1%)	193 (80.4%)
	**Former smoker**	20 (5.1%)	16 (6.7%)
	**Current smoker**	42 (10.7%)	31 (12.9%)
**Maternal age (years)** [a]		26.3 ± 4.4	26.2 ± 4.9
**Maternal BMI (kg/m^2)** [a]		21.3 ± 2.5	21.5 ± 2.5
**Maternal BMI status** [b]	**Underweight**	39 (10.0%)	23 (9.5%)
	**Normal weight**	270 (69.0%)	161 (66.8%)
	**Overweight/obesity**	82 (21.0%)	57 (23.7%)
**Maternal smoking during pregnancy**		3 (0.8%)	2 (0.8%)
**Maternal education** [c]	**Less than high school**	272 (80.5%)	170 (82.5%)
	**High school or greater**	66 (19.5%)	36 (17.5%)
**Paternal education** [c]	**Less than high school**	242 (71.6%)	142 (69.0%)
	**High school or greater**	96 (28.4%)	64 (31.1%)
**Family income (baht per month)** [d]		2430 [1500, 3900]	2700 [1800, 4400]

Family income is reported as the median [Q1, Q3], other continuous data as the means ± standard deviations, and categorical data as *n* (%).
BMI, body mass index; DXA, whole-body dual-energy X-ray absorptiometry
[a] Maternal age and BMI were recorded at the first antenatal visit (median of 14 weeks of gestation) in the original study in 1989–1990.
[b] Classified according to the Asia-Pacific criteria (Tham et al., Obes Rev 2023;24:e13520): underweight, BMI<18.5 kg/m^2; normal weight, BMI ≥18.5 to <23 kg/m^2; overweight, BMI ≥23 to <25 kg/m^2; and obesity, BMI ≥25 kg/m^2.
[c] There were missing data on the highest levels of education; hence, the available sample sizes for the Included and Excluded groups were 338 (86.4%) and 206 (85.5%) for maternal education, respectively, and 338 (86.4%) and 206 (85.5%) for paternal education.
[d] Income recorded at the time of maternal recruitment to the original study in 1989–1990 (i.e., not adjusted for inflation); the available sample sizes were 326 (83.4%) and 202 (83.8%) for the Included and Excluded groups, respectively.
*p<0.05, **p<0.01 and ***p<0.001 for a difference between the two groups, assessed using two-sample *t*-tests.

Supplementary Table 3. Linear associations between maternal BMI early in pregnancy and body composition in the young adult offspring (*n*=391) assessed using DXA scans.

Body composition parameter	Model	β (95% CI)	*P*-value
**Total body fat mass (kg)**	Unadjusted	0.39 (0.14, 0.65)	**0.003**
	Adjusted	0.46 (0.20, 0.72)	**0.001**
**Fat mass index (kg/m²)**	Unadjusted	0.14 (0.04, 0.24)	**0.006**
	Adjusted	0.17 (0.07, 0.26)	**<0.001**
**Total body lean mass (kg)**	Unadjusted	0.46 (0.11, 0.82)	**0.011**
	Adjusted	0.44 (0.19, 0.70)	**0.001**
**Lean mass index (kg/m²)**	Unadjusted	0.15 (0.06, 0.25)	**0.001**
	Adjusted	0.16 (0.08, 0.24)	**<0.001**
**Total body fat (%)**	Unadjusted	0.23 (-0.07, 0.53)	0.14
	Adjusted	0.30 (0.07, 0.52)	**0.009**
**Android-to-gynoid-fat ratio**	Unadjusted	0.002 (-0.004, 0.007)	0.50
	Adjusted	0.003 (-0.002, 0.008)	0.28

BMI, body mass index; DXA, whole-body dual-energy X-ray absorptiometry.
Unadjusted data are the β coefficients and 95% confidence intervals (CI) derived from general linear regression models (GLM). The adjusted GLM also included as independent variables maternal age, parity, gestational age at birth, and offspring sex.
p-values shown in bold are statistically significant at $p<0.05$.

Supplementary Table 4. Anthropometry and BMI status in the young adult offspring (*n*=391) who underwent DXA scans stratified by their mother's BMI status in pregnancy in the Chiang Mai Low Birth Weight Study.

OFFSPRING ANTHROPOMETRY		MATERNAL BMI STATUS		
		Underweight	Normal weight	Overweight/obesity
n		39 (10.0%)	270 (69.0%)	82 (21%)
**BMI status [A]**	**Underweight**	16 (41.0%)	78 (28.9%)	15 (18.3%)
	**Normal weight**	18 (46.2%)	133(49.3%)	38 (46.3%)
	**Overweight**	3 (7.7%)	25 (9.3%)	10 (12.2%)
	**Obesity**	2 (5.1%)	34 (12.6%)	19 (23.2%)
**BMI status (binary) [B]**	**Underweight/normal weight**	34 (87.2%)	211(78.2%)	53 (64.6%)
	**Overweight/obesity**	5 (12.8%)	59 (21.9%)	29 (35.4%)

Data are *n* (%).
BMI, body mass index; DXA, whole-body dual-energy X-ray absorptiometry.
BMI was obtained at the first antenatal visit (median of 14 weeks of gestation) and classified according to the Asia-Pacific criteria (Tham et al. Obes Rev 2023;24:e13520): underweight, BMI<18.5 kg/m^2; normal weight, BMI ≥18.5 to <23 kg/m^2; overweight, BMI ≥23 to <25 kg/m^2; and obesity, BMI ≥25 kg/m^2.
[A] p=0.039 and [B] p=0.012 for the differences between maternal BMI groups, assessed with Fisher's exact tests.

Supplementary Table 5. Anthropometry and BMI status in the young adult offspring who did not undergo DXA scans (*n*=241) stratified by their mother's BMI status in pregnancy in the Chiang Mai Low Birth Weight Study.

OFFSPRING ANTHROPOMETRY		MATERNAL BMI STATUS		
		**Underweight**	**Normal weight**	**Overweight/obesity**
n		23 (9.5%)	161 (66.8%)	57 (23.7%)
**BMI status** [A]	**Underweight**	10 (43.5%)	42 (26.1%)	8 (14.0%)
	**Normal weight**	12 (52.2%)	75 (46.6%)	22 (38.6%)
	**Overweight**	–	11 (6.8%)	9 (15.8%)
	**Obesity**	1 (4.3%)	33 (20.5%)	18 (31.6%)
**BMI status (binary)** [B]	**Underweight/normal weight**	22 (95.7%)	117 (72.7%)	30 (52.6%)
	**Overweight/obesity**	1 (4.3%)	44 (27.3%)	27 (47.4%)

Data are *n* (%).

BMI, body mass index; DXA, whole-body dual-energy X-ray absorptiometry.

BMI was obtained at the first antenatal visit (median of 14 weeks of gestation) and classified according to the Asia-Pacific criteria (Tham et al. Obes Rev 2023;24:e13520): underweight, BMI<18.5 kg/m^2; normal weight, BMI ≥18.5 to <23 kg/m^2; overweight, BMI ≥23 to <25 kg/m^2; and obesity, BMI ≥25 kg/m^2.

[A] p=0.004 and [B] p<0.001 for the differences between maternal BMI groups, assessed with Fisher's exact tests.
